# Efficacy and safety of Programmed Death Ligand 1 inhibitors *versus* Programmed Death 1 inhibitors in the first-line treatment of advanced non-small cell lung cancer: a meta-analysis of randomized controlled trials

**DOI:** 10.7717/peerj.21402

**Published:** 2026-07-07

**Authors:** Cui Dang, Jingzhang Li, Hong Chen, Zoujie Luo

**Affiliations:** 1 Department of Endocrinology, The First Affiliated Hospital of Guangxi Medical University, Nanning, Guangxi, China; 2 Department of Oncology, Liuzhou People’s Hospital Affiliated to Guangxi Medical University, Liuzhou, Guangxi, China; 3 Department of Nephrology, The Second People’s Hospital of Qinzhou, Guangxi, China

**Keywords:** PD-L2 inhibitors, PD-1 inhibitors, Advanced non-small cell lung cancer, Efficacy, Safety

## Abstract

**Background:**

With the increasing use of immune checkpoint inhibitors (ICIs) in the first-line treatment of driver mutation-negative non-small cell lung cancer (NSCLC), we conducted a systematic review and meta-analysis to compare efficacy and adverse effects (AEs) between PD-L1 inhibitors and PD-1 inhibitors.

**Methods:**

We searched PubMed, Web of Science, Embase, and the Cochrane Library to identify randomized controlled trials (RCTs) related to the first-line treatment of NSCLC with ICIs alone or in combination with chemotherapy. The primary outcomes were overall survival (OS), progression-free survival (PFS), and AEs.

**Results:**

In total, 28 RCTs involving 14,758 patients were included. Compared with Programmed Death 1 (PD-1) inhibitors plus chemotherapy, Programmed Death Ligand 1 (PD-L1) inhibitors plus chemotherapy were associated with worse OS (hazard ratio (HR) = 1.26; 95% confidence interval (CI) [1.13–1.41]; *P* < 0.001) and PFS (HR = 1.21; 95% CI [1.06–1.38]; *P* = 0.005). The objective response rate (ORR) did not differ between PD-1 inhibitors plus chemotherapy and PD-L1 inhibitors plus chemotherapy (odds ratio (OR) = 0.91; 95% CI [0.78–1.05], *P* = 0.205), and AE rates were similar between these groups. Regarding monotherapy, no difference in OS, PFS, or ORR was observed between PD-L1 and PD-1 inhibitors. The incidence of AEs leading to treatment termination and grade ≥3 AEs was lower for PD-L1 inhibitors than PD-1 inhibitors (risk ratio (RR) = 0.55; 95% CI [0.32–0.95]; *P* = 0.03; RR = 0.76; 95% CI [0.60–0.96]; *P* = 0.021).

**Conclusions:**

The combination of PD-1 inhibitors and chemotherapy may provide a significant OS and PFS benefit relative to PD-L1 inhibitors plus chemotherapy in NSCLC and a similar safety profile. Meanwhile, PD-L1 inhibitor monotherapy appears less likely to result in treatment termination or grade ≥3 AEs than PD-1 inhibitor monotherapy.

## Introduction

Lung cancer is the most common cancer globally, accounting for one-eighth of all cancers (Bray et al., 2024). Non-small cell lung cancer (NSCLC) is the most common subtype, accounting for approximately 85% of all cases of lung cancer (Molina et al., 2008). Over recent decades, chemotherapy has become the primary treatment for NSCLC. In 2000, the ECOG1594 study established platinum-based chemotherapy as the standard treatment for patients with NSCLC. However, the efficacy of chemotherapy has plateaued, achieving overall response rates (ORRs) of approximately 15%–22%, median progression-free survival (PFS) of approximately 3–5 months, and median overall survival (OS) of approximately 8 months (Schiller et al., 2002).

In 2009, the IPASS study revealed that epidermal growth factor  receptor (EGFR)-tyrosine kinase inhibitor treatment was effective in patients with EGFR-mutated advanced NSCLC, and the era of targeted precision therapy has gradually emerged (Fukuoka et al., 2011). Although some patients benefit from molecular targeted therapy, its efficacy is limited to patients with gene mutations such as *anaplastic lymphoma kinase* rearrangements, *EGFR* mutations, *ROS proto-oncogene receptor tyrosine kinase 1* rearrangements, and *BRAF* V600E mutations. However, these mutations are found in only 30% of patients with NSCLC (Arbour & Riely, 2019). Therefore, there is an urgent need for safer and more effective treatments for advanced NSCLC.

In recent years, immunotherapy has produced remarkable achievements in the treatment of advanced NSCLC. Immune checkpoint inhibitors (ICIs) work by releasing the inhibitory brake of T cells, leading to the activation of immune responses against cancers (Bagchi, Yuan & Engleman, 2021). The KEYNOTE-024 study was the first to demonstrate that pembrolizumab can improve survival compared with chemotherapy in patients with high PD-L1 expression, with OS reaching 30 months for pembrolizumab and 14.2 months for chemotherapy (Reck et al., 2016). Similarly, the KEYNOTE-042 study reported that PD-1 inhibitors performed better than chemotherapy in PD-L1-positive, driver mutation-negative advanced NSCLC (De Castro Jr. et al., 2023). Meanwhile, the KEYNOTE-189 and KEYNOTE-407 studies found that pembrolizumab combined with chemotherapy was more effective than chemotherapy alone in patients with driver-negative advanced NSCLC, regardless of PD-L1 expression (Garassino et al., 2023; Novello et al., 2023). In addition, the IMpower130 study confirmed that the PD-L1 inhibitor atezolizumab plus chemotherapy was more beneficial than chemotherapy alone in advanced non-squamous NSCLC (West et al., 2019). In recent years, immunosuppressants have achieved positive results in patients with mutation-negative lung cancer and in those with *EGFR* mutation-positive lung cancer. The ORIENT-31 trial found that compared with chemotherapy alone, sintilimab combined with chemotherapy or sintilimab plus IBI305 and chemotherapy provided a PFS benefit in the second-line treatment of patients with *EGFR*-mutated lung cancer (Lu et al., 2023). Additionally, multiple studies have confirmed that the use of immunotherapy during the perioperative period provides clinical benefits to patients. The RATIONALE-315 study reported that tislelizumab significantly improved the major pathological response rate and event-free survival *versus* placebo (Yue et al., 2025). With ongoing research and development, new immunotherapeutic drugs continue to emerge, including bispecific antibody immunotherapeutics such as ivonescimab. The HARMONi-2 study demonstrated that ivonescimab significantly improved PFS compared with pembrolizumab in advanced PD-L1-positive non-small cell lung cancer (Xiong et al., 2025).

The 2026 meta-analysis showed, without a doubt, that PD-1/PD-L1 inhibitors combined with chemotherapy resulted in superior OS and PFS compared with chemotherapy alone (Rao et al., 2025). The most recent systematic review comparing PD-1 inhibitors *versus* PD-L1 inhibitors in non-small cell lung cancer was published in February 2025 and included only nine randomized controlled trials. This study specifically examined the differences in efficacy and safety between PD-1 inhibitors plus chemotherapy and PD-L1 inhibitors plus chemotherapy in squamous NSCLC  (Liu et al., 2025). However, the differences between these two classes of inhibitors in non-small cell lung cancer remain unknown, as do the differences between PD-1 inhibitor monotherapy and PD-L1 inhibitor monotherapy. Furthermore, an increasing number of randomized controlled trials have subsequently reported their results. Because of the growing number of clinical trials investigating ICIs for the treatment of advanced NSCLC and the increasing use of ICIs in clinical practice, the differences in efficacy and safety between PD-L1 and PD-1 inhibitors have also received increasing attention. Therefore, we conducted a systematic review to compare the efficacy and safety of PD-L1 and PD-1 inhibitors in advanced NSCLC for clinical reference.

## Methods

### Study design

A meta-analysis of randomized controlled trials (RCTs) that investigated the efficacy and safety of PD-L1 or PD-1 inhibitors alone or in combination with chemotherapy in the first-line treatment of patients with advanced, treatment-naïve NSCLC was conducted.

### Literature search strategy

Searches for relevant studies published before December 31, 2025, were performed in PubMed, Web of Science, Embase, and Cochrane Library. The main search terms were as follows: “NSCLC” OR “Carcinoma, Non-Small-Cell Lung” OR “Non small Cell Lung Cancer” and “Immune Checkpoint Inhibitor” OR “Immune Checkpoint Blockers” OR “Immune Checkpoint Blockade” OR “Immune Checkpoint Inhibition” “Programmed Death Ligand 1 Inhibitors” OR “Programmed Cell Death Protein 1 Inhibitors” OR “PD-L1” OR “PD-1” OR “pembrolizumab” OR “nivolumab” OR “atezolizumab” OR “durvalumab” OR “toripalimab” OR “sintilimab” OR “camrelizumab” OR “tislelizumab” OR “sugemalimab” OR “penpulimab” OR “serplulimab” and “Randomized Controlled Trials” OR “Controlled Clinical Trials” OR “RCT”.

### Study selection

Studies meeting the following criteria were included: histological confirmation of treatment-naïve unresectable advanced NSCLC; the intervention group received PD-1/PD-L1 inhibitors alone or PD-1/PD-L1 inhibitors plus chemotherapy and teg control group received chemotherapy alone; the outcomes included OS, PFS, ORR, and AEs; and the articles were published in English language. When multiple articles reporting the same trial were identified, only the most recent publication was included.

The exclusion criteria were as follows: meta-analyses, reviews, editorials, case reports, and letters; prospective or retrospective observational cohort studies; patients received previous systemic treatment; patients received spontaneous anti-CTLA-4 treatment or chemoradiotherapy; and duplicate articles.

### Data extraction

Two investigators (DC and CH) independently searched the included studies and collected clinical information, including first author, publication year, patient characteristics, treatment protocol, age, sample size, OS, PFS, ORR, and treatment-related severe AEs. They compared their collected data in cases of discrepancies and consulted a third investigator (LJZ) to resolve disagreements.

This review was conducted according to the Preferred Reporting Items for Systematic Review and Meta-Analyses statement (PROSPERO; Registration No. CRD42024496951).

### Quality assessment

Two researchers independently evaluated the risk of bias for the included studies using the Cochrane Risk of bias tool. Disagreements were resolved by a third researcher. We assessed the risk of bias according to the following domains: random sequence generation, allocation concealment, blinding implementation, completeness of data, selective reporting, and other sources of bias.

### Statistical analysis

We used Review Manager version 5.3 to perform statistical analyses. In indirect comparisons, the *Q*-test and *I*^2^ statistic were used to assess heterogeneity among the included studies. A random-effects model was used when significant heterogeneity was identified (*I*^2^ > 50% and *P* < 0.10); otherwise, a fixed-effects model was used. The hazard ratio (HR) and 95% confidence interval (CI) were determined to estimate the aggregate estimates for OS and PFS. The odds ratio (OR) and 95% CI were used to estimate differences in ORR and toxicity. Begg’s test and Egger’s test were used to estimate publication bias. *P* < 0.05 denoted statistical significance. In indirect comparisons, we performed adjusted indirect comparison using the Bucher method with the following formula (Bucher et al., 1997): log HR_AB_ = log HR_AC_ − log HR_BC_. The formula for standard error (SE) of log HR was SE (log HR_AB_) = $\sqrt{\mathrm{SE}(\mathrm{log}~{\mathrm{HR}}_{\mathrm{AC}})^{2}+\mathrm{SE}(\mathrm{log}~{\mathrm{HR}}_{\mathrm{BC}})^{2}}$. The risk ratio (RR) was calculated in a similar manner.

## Results

### Study selection and characteristics

**Figure 1:**
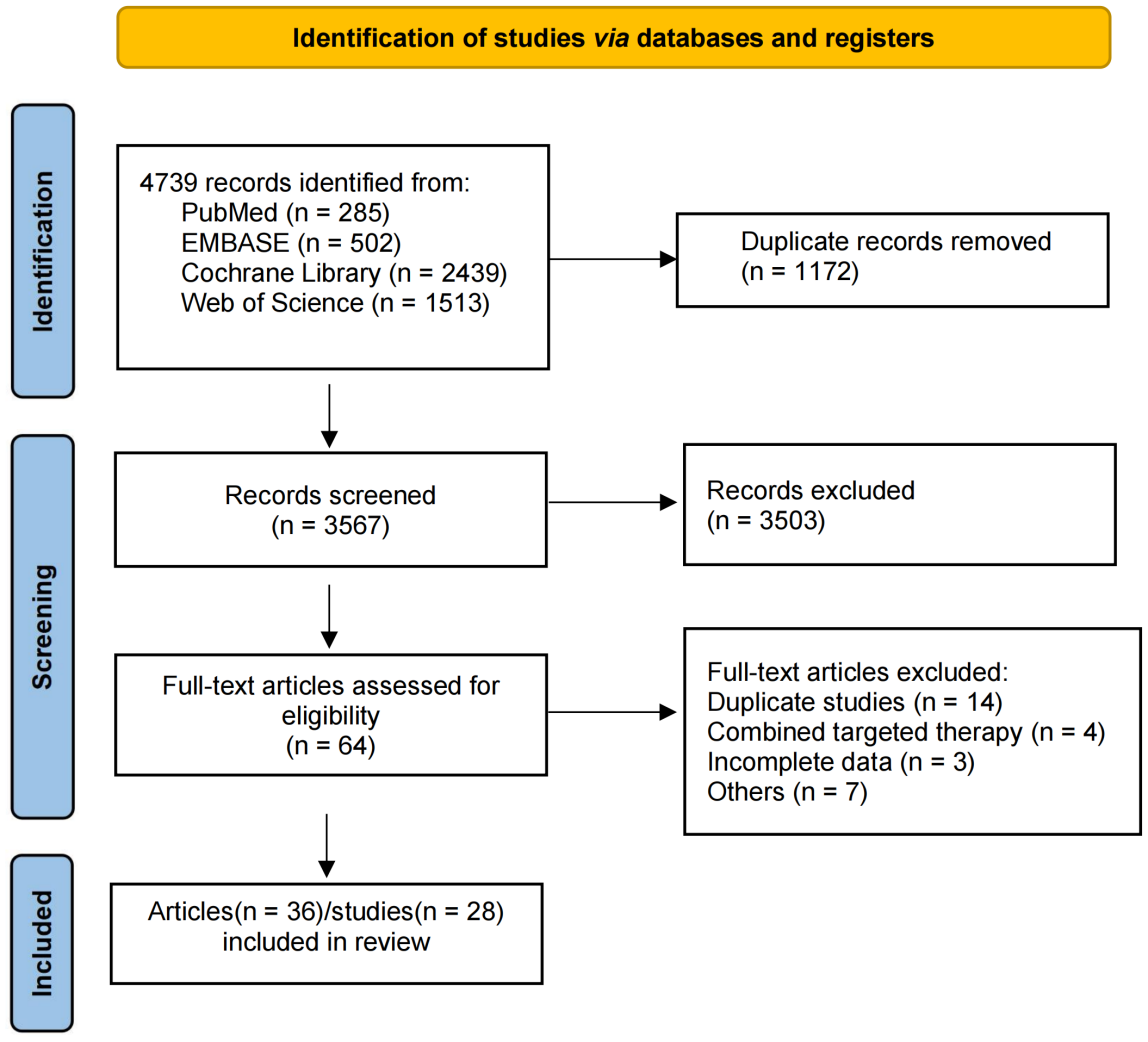
Identification of studies *via* databases and registers. The number of articles included and excluded at each step of the screening and the reasons for them. Eventually, 28 clinical studies and 36 articles were included, among which eight were extended survival followups.

**Figure 2:**
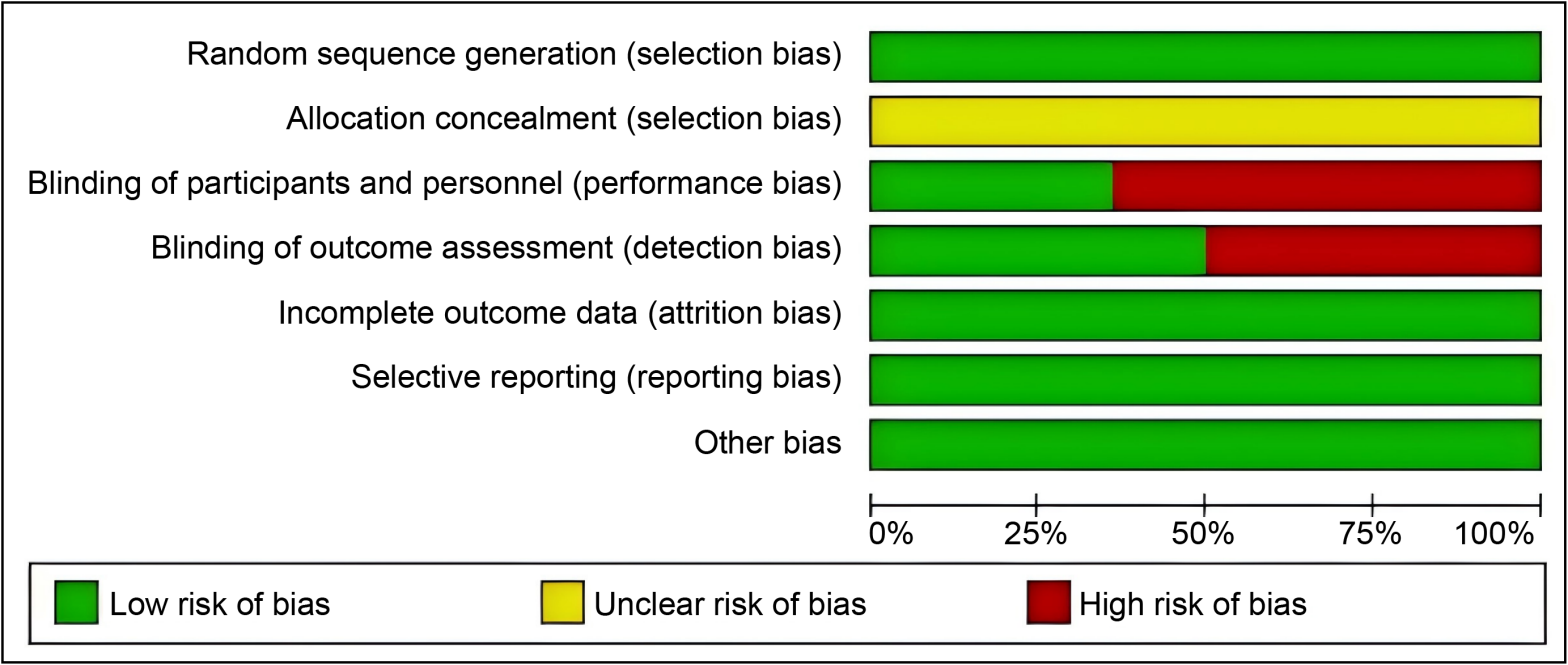
The risk of bias assessment. The bias assessment shows that the risk of bias of the articles included in this article is relatively low.

**Figure 3:**
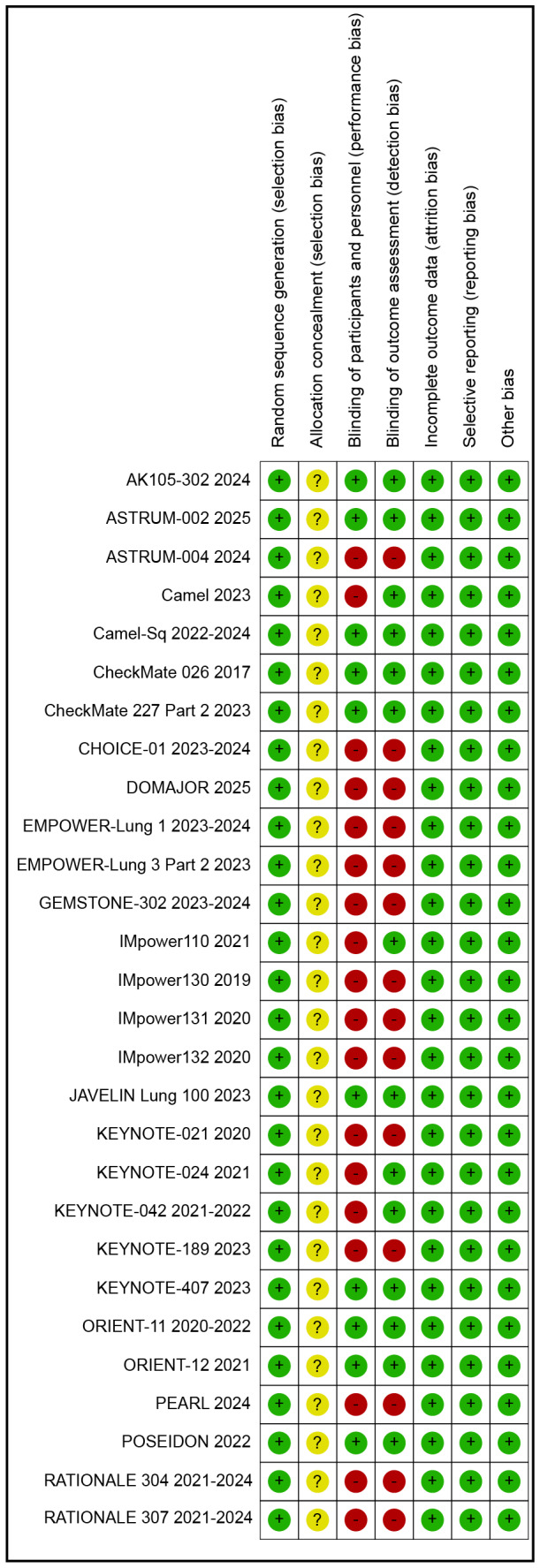
Risk of bias assessment results using the Cochrane risk of bias tool. The bias assessment shows that the risk of bias of the articles included in this paper is relatively low.

In total, 4,739 potentially relevant studies were identified in the databases. After screening the abstracts and full texts of the studies, 4,711 studies were excluded, and 28 high-quality trials involving 14,758 patients were included in this meta-analysis (De Castro Jr. et al., 2023; Garassino et al., 2023; Novello et al., 2023; West et al., 2019; Nishio et al., 2021; Awad et al., 2021; Lu et al., 2021; Zhang et al., 2022; Yang et al., 2020; Jotte et al., 2020; Zhou et al., 2021; Wang et al., 2021; Ren et al., 2022; Carbone et al., 2017; Borghaei et al., 2023; Reck et al., 2021; De Castro Jr. et al., 2021; Jassem et al., 2021; Wang et al., 2023; Makharadze et al., 2023; Reck et al., 2024; Johnson et al., 2023; Özgüroğlu et al., 2023; Zhou et al., 2023a; Zhou et al., 2023b; Ma et al., 2024; Zhou et al., 2024b; Wang et al., 2024; Zhong et al., 2024b; Kilickap et al., 2024; Zhou et al., 2024c; Lu et al., 2025; Zhong et al., 2024a; Zhou et al., 2024a; Wang et al., 2026; Laktionov et al., 2025). Figure 1 presents the complete screening process and selection procedures. The included studies were RCTs published between 2017 and 2025, and they included 26 phase III trials and two phase II trials. These studies included eight trials that used PD-L1 inhibitors, including three trials that used PD-L1 inhibitors alone and five trials that utilized PD-L1 inhibitors combined with chemotherapy. Patients were treated with PD-1 inhibitors in 20 trials, including four trials that used PD-1 inhibitor monotherapy and 16 studies that used PD-1 inhibitors plus chemotherapy. The main characteristics of the 28 trials are presented in Tables 1 and 2. The risk of bias assessment is presented in Figs. 2 and 3.

**Table 1:** Baseline characteristics of randomized controlled trials included in the meta-analysis (*n* = 14, 758). The 28 clinical studies included in this research.

Trial name	NCT-ID	Year	Arm	*N*	Study phase	Stage	Follow-up (m)	Squamous (%)	Non-squamous (%)	Median age	Males (%)	Smoke (%)	ECOG 0 (%)	ECOG 1 (%)	Brain metastases (%)	Tumor PD-L1 expression <1 (%)
IMpower110 (Jassem et al., 2021)	02409342	2021	Atezolizumab	277	3	IV	30.0	30.7	69.3	64.0	70.8	86.6	35.0	65.0	NA	0.0
			Chemotherapy	277		IV	30.0	30.3	69.7	65.0	69.7	87.3	37.2	62.8	NA	0.0
JAVELIN Lung 100 (Reck et al., 2024)	02576574	2023	Avelumab	366	3	IV	48.8	33.9	66.1	64	77	88.3	33.3	66.4	7.9	0
			Chemotherapy	526		IV	45.6	34.6	65.4	63	72.4	85.4	36.5	63.1	10.6	0
PEARL (Lu et al., 2025)	03003962	2024	Durvalumab	335	3	IV	53.7	39.4	60.6	61	80	78.8	20.3	79.7	14.6	0
			Chemotherapy	334		IV	47.5	39.8	60.2	63.4	80.5	78.1	23.7	76.3	13.2	0
CHECKMATE 026 (Carbone et al., 2017)	02041533	2017	Nivolumab	271	3	IV or recurrent	13.5	24	76	63	68	88	31	68	12	0
			Chemotherapy	270		IV or recurrent	13.5	24	76	65	55	87	34	64	13	0
KEYNOTE-024 (Reck et al., 2021)	02142738	2021	Pembrolizumab	154	3	IV	59.9	18.8	81.2	64.5	59.7	96.8	35.1	64.3	11.7	0
			Chemotherapy	151		IV	59.9	17.9	82.1	66	62.9	87.4	35.1	64.9	6.6	0
KEYNOTE-042 (De Castro Jr. et al., 2023; De Castro Jr. et al., 2021)	02220894	2021/2022	Pembrolizumab	637	3	Locally advanced/ metastatic	61.1	38	62	63	70.6	77.7	30.9	68.9	5.5	0
			Chemotherapy	637		Locally advanced/ metastatic	61.1	39.1	60.9	63	71	78	30.1	69.9	5.5	0
EMPOWER-Lung 1 (Özgüroğlu et al., 2023; Kilickap et al., 2024)	03088540	2023/2024	Cemiplimab	357	3	IIIB to IV	57.3	45	55	63	88	100	27	73	12	NA
			Chemotherapy	355		IIIB to IV	57.3	43	57	64	83	100	27	73	11	NA
IMpower130 (West et al., 2019)	02367781	2019	Atezolizumab + Chemotherapy	451	3	IV	18.5	0	100	64	59	89	42	58	NA	52
			Chemotherapy	228			19.2	0	100	65	59	92	40	60	NA	53
IMpower132 (Nishio et al., 2021)	01633970	2020	Atezolizumab + Chemotherapy	292	3	IV	28.4	0	100	64	65.8	87.3	43.2	56.8	NA	50
			Chemotherapy	286			28.4	0	100	63	67.1	89.5	40.1	59.9	NA	44.6
IMpower131 (Jotte et al., 2020)	02367794	2020	Atezolizumab + Chemotherapy	343	3	IV	26.8	100	0	65	81.6	90.7	33.5	66.2	NA	46.6
			Chemotherapy	340			24.8	100	0	65	81.6	92.9	32.4	67.4	NA	50.3
POSEIDON (Johnson et al., 2023)	03164616	2022	Durvalumab + Chemotherapy	338	3	IV	34.9	37.9	61.8	64.5	74.9	75.1	32.2	67.8	8.3	33.4
			Chemotherapy	337			34.9	36.2	63.5	64	73.6	76.3	35.3	64.4	13.4	38.6
GEMSTONE-302 (Zhou et al., 2023b; Zhou et al., 2024c)	03789604	2023/2024	Sugemalimab + Chemotherapy	320	3	IV	43.5	40.3	59.7	62	79.4	72.5	18.4	81.6	15.6	38.8
			Chemotherapy	159			43	39.6	60.4	64	81.1	74.8	15.7	84.3	10.7	40.3
CameL (Zhou et al., 2023a)	03134872	2023	Camrelizumab + Chemotherapy	205	3	IIIB to IV	24.2	0	100	59	71.2	62	23.4	76.6	4.9	23.9
			Chemotherapy	207			17.8	0	100	61	72	62.8	17.4	82.6	2.4	33.5
KEYNOTE-021 (Awad et al., 2021)	02039674	2020	Pembrolizumab + Chemotherapy	60	2	IIIB to IV	49.4	0	100	62.5	37	75	40	58	20	35
			Chemotherapy	63			49.4	0	100	66	41	86	46	54	11	37
RATIONALE 304 (Lu et al., 2021; Ma et al., 2024)	03663205	2021/2024	Tislelizumab + Chemotherapy	223	3	IIIB to IV	49.4	0	100	60	75.3	65.9	24.2	75.8	4.9	43
			Chemotherapy	111			49.4	0	100	61	71.2	59.4	21.6	78.4	6.3	43.2
ORIENT-11 (Zhang et al., 2022; Yang et al., 2020)	03607539	2020/2022	Sintilimab + Chemotherapy	266	3	IIIB to IV	30.8	0	100	61	76.7	64.3	28.6	71.4	13.5	32
			Chemotherapy	131			30.8	0	100	61	75.6	66.5	26	74	16.8	33.6
KEYNOTE-189 (Garassino et al., 2023)	02578680	2023	Pembrolizumab + Chemotherapy	410	3	IV	64.6	0	100	65	62.0	88.3	45.1	53.9	17.8	31
			Chemotherapy	206			64.6	0	100	63.5	52.9	87.9	38.3	61.2	17.0	30.6
ORIENT-12 (Zhou et al., 2021)	03629925	2021	Sintilimab + Chemotherapy	179	3	IIIB to IV	12.9	100	0	64	91.1	86.6	16.8	83.2	NA	33
			Chemotherapy	178			12.9	100	0	62	92.1	82.6	12.4	87.6	NA	35.4
RATIONALE 307 (Wang et al., 2021; Wang et al., 2024)	03594747	2021/2024	Tislelizumab + Chemotherapy	120	3	IIIB to IV	50.3	100	0	60	89.2	80.0	25.8	74.2	1.7	40
			Chemotherapy	121			50.3	100	0	62	91.7	81.0	26.4	73.6	0.8	40.5
CameL-Sq (Ren et al., 2022; Zhou et al., 2024b)	03668496	2022/2024	Camrelizumab + Chemotherapy	193	3	IIIB to IV	53.5	100	0	64	93	84	20	80	2	47
			Chemotherapy	196			53.5	100	0	62	92	80	22	78	2	49
KEYNOTE-407 (Novello et al., 2023)	02775435	2023	Pembrolizumab + Chemotherapy	278	3	IV	56.9	100	0	65	79.1	92.1	26.3	73.7	7.2	34.2
			Chemotherapy	281			56.9	100	0	65	83.6	93.2	32	68	8.2	35.2
CheckMate 227 Part 2 (Borghaei et al., 2023)	02477826	2023	Nivolumab + Chemotherapy	377	3	IV/recurrent	36	28	72	63	70	84	35	64	11	43
			Chemotherapy	378			36	28	72	64	70	79	28	71	9	43
CHOICE-01 (Wang et al., 2023; Zhong et al., 2024b)	03856411	2023/2024	Toripalimab + Chemotherapy	309	3	IIIB to IV	21.2	47.6	52.4	63	79.9	68.9	21.4	78.6	1.6	35
			Chemotherapy	156			21.2	46.8	53.2	61	83.3	68.6	23.1	76.9	0	34
EMPOWER-Lung 3 Part 2 (Makharadze et al., 2023)	03409614	2023	Cemiplimab + Chemotherapy	312	3	IIIB to IV	28.3	42.6	57.4	63	85.9	86.2	16.3	83	NA	30.4
			Chemotherapy	154			28.7	43.5	56.5	63	79.9	84.4	11.7	87	NA	28.6
AK105-302 (Zhong et al., 2024a)	03866993	2024	Penpulimab + Chemotherapy	175	3	IIIB to IV	24.7	100	0	60.9	93	89	26	74	3	34
			Chemotherapy	175			24.7	100	0	61.9	93	87	24	76	2	33
ASTRUM-004 (Zhou et al., 2024a)	04033354	2024	Serplulimab + Chemotherapy	358	3	IIIB to IV	31.1	98.6	1.5	63	89.7	86	18.2	81.8	37.7	5.6
			Chemotherapy	179			31.1	97.6	2.3	63	93.3	88.8	14.5	85.5	38	10.1
ASTRUM-002 (Wang et al., 2026)	03952403	2025	Serplulimab + Chemotherapy	214	3	IIIB to IV	23.1	0	100	62	73	67	28	72	19	39
			Chemotherapy	210			23	0	100	61	74	67	28	72	19	32
DOMAJOR (Laktionov et al., 2025)	03912389	2025	Prolgolimab + Chemotherapy	143	3	IV	17.9	0	100	62	66.4	69.2	28	72	NA	39.9
			Chemotherapy	149			17.9	0	100	62	71.8	73.2	24.8	75.2	NA	40.3

**Notes.**

NCT-ID National Clinical Trial Identifier

PD-L1 inhibitors: Atezolizumab, Durvalumab, Sugemalimab, Avelumab; PD-1 inhibitors: Nivolumab, Pembrolizumab, Cemiplimab, Camrelizumab, Tislelizumab, Sintilimab, Nivolumab, Toripalimab, Penpulimab, Serplulimab, Prolgolimab. There are seven studies comparing immunotherapy monotherapy with chemotherapy, of which three (Jassem et al., 2021; Reck et al., 2024; Lu et al., 2025) are PD-L1 inhibitors *versus* chemotherapy, and four (Carbone et al., 2017; Reck et al., 2021; De Castro Jr. et al., 2023; De Castro Jr. et al., 2021; Özgüroğlu et al., 2023; Kilickap et al., 2024) are PD-1 inhibitors *versus* chemotherapy. 21 studies comparing immunotherapy combined with chemotherapy *versus* chemotherapy alone. Among them, five (West et al., 2019; Nishio et al., 2021; Jotte et al., 2020; Johnson et al., 2023; Zhou et al., 2023a; Zhou et al., 2023b; Zhou et al., 2024a; Zhou et al., 2024b; Zhou et al., 2024c) are PD-L1 inhibitors combined with chemotherapy *versus* chemotherapy, and 16 (Zhou et al., 2023a; Zhou et al., 2023b; Awad et al., 2021; Lu et al., 2021; Ma et al., 2024; Zhang et al., 2022; Yang et al., 2020; Garassino et al., 2023; Zhou et al., 2021; Wang et al., 2021; Wang et al., 2024; Ren et al., 2022; Novello et al., 2023; Borghaei et al., 2023; Wang et al., 2023; Zhong et al., 2024b; Makharadze et al., 2023; Zhong et al., 2024a; Zhou et al., 2024a; Zhou et al., 2024b; Zhou et al., 2024c; Wang et al., 2026; Laktionov et al., 2025) are PD-1 inhibitors combined with chemotherapy *versus* chemotherapy.

**Table 2:** Outcome of included randomized controlled trials. Outcome information of the 28 clinical studies included in this research.

Trial name	Grade 3–5 TRAE (%)	Any grade TRAE *N* (%)	Leading to discontinuation *N* (%)	Leading to death *N* (%)	ORR (%)	OS (m)	PFS (m)	HR for PFS 95% CI	HR for OS 95% CI
IMpower110 (Jassem et al., 2021)	41 (14.3)	180 (62.9)	21 (7.3)	2 (0.7)	31.4	18.9	5.8	0.72 (0.60–0.86)	0.84 (0.68-1.03)
	118 (45.3)	224 (85.2)	45 (17.1)	3 (1.1)	32.1	14.7	5.6		
JAVELIN Lung 100 (Reck et al., 2024)	60 (16.6)	243 (67.3)	44 (12.2)	3 (0.8)	25.4	20.1	8.4	0.71 (0.54–0.93)	0.85 (0.67–1.09)
	230 (46)	430 (86)	76 (15.2)	6 (1.2)	31.0	14.9	5.6			
PEARL (Lu et al., 2025)	52 (15.5)	195 (58.2)	21 (6.3)	8 (2.4)	37.6	14.6	5.4	0.77 (0.65–0.92)	0.84 (0.71–0.99)
	150 (45.9)	281 (85.9)	41 (12.5)	3 (0.9)	37.4	12.8	4.8		
CHECKMATE 026 (Carbone et al., 2017)	47 (18)	190 (71)	26 (9.7)	2 (0.7)	26	13.7	4.2	1.19 (0.97–1.46)	1.08 (0.87–1.34)
	133 (51)	243 (92)	35 (13.3)	3 (1.1)	33	13.8	5.8		
KEYNOTE-024 (Reck et al., 2021)	48 (31.2)	118 (76.6)	21 (13.6)	2 (1.3)	46.1	26.3	7.7	0.50 (0.39–0.65)	0.62 (0.48–0.81)
	80 (53.3)	135 (90)	16 (10.7)	3 (2)	31.1	13.4	5.5		
KEYNOTE-042 (De Castro Jr. et al., 2023; De Castro Jr. et al., 2021)	120 (18.9)	406 (63.8)	58 (9.1)	13 (2)	27.3	16.4	5.6	1.03 (0.91–1.16)	0.79 (0.70–0.89)
	257 (41.8)	555 (90.2)	59 (9.6)	14 (2.3)	26.7	12.1	6.8		
EMPOWER-Lung 1 (Özgüroğlu et al., 2023; Kilickap et al., 2024)	65 (18)	223 (63)	26 (7)	10 (3)	46.5	26.1	8.1	0.5 (0.41–0.61)	0.585 (0.48–0.72)
	137 (40)	310 (90)	15 (4)	7 (2)	20.6	13.3	5.3		
IMpower130 (West et al., 2019)	354 (75)	455 (96.2)	125 (26.4)	8 (1.7)	49.2	18.6	7	0.64 (0.54–0.77)	0.79 (0.64–0.98)
	141 (61)	215 (92.7)	51 (22)	1 (0.43)	31.9	13.9	5.5		
IMpower132 (Nishio et al., 2021)	170 (58.4)	266 (91.4)	83 (28.5)	NA	47	17.5	7.6	0.60 (0.49–0.72)	0.86 (0.71–1.06)
	118 (43)	240 (87.6)	50 (18.2)	NA	32	13.6	5.2		
IMpower131 (Jotte et al., 2020)	231 (69.2)	316 (94.6)	102 (30.5)	NA	49.7	14.2	6.3	0.71 (0.60–0.85)	0.88 (0.73–1.05)
	195 (58.4)	303 (90.7)	58 (17.4)	NA	41.0	13.5	5.6		
POSEIDON (Johnson et al., 2023)	149 (44.6)	296 (88.60)	47 (14.1)	7 (2.1)	41.5	13.3	5.5	0.74 (0.62–0.89)	0.86 (0.72–1.02)
	148 (44.40	298 (89.50)	33 (9.9)	8 (2.4)	24.4	11.7	4.8		
GEMSTONE-302 (Zhou et al., 2023b; Zhou et al., 2024c)	NA	NA	NA	11 (3.4)	63.4	25.2	9.0	0.49 (0.39–0.60)	0.68 (0.54–0.85)
	NA	NA	NA	2 (1.3)	40.3	16.9	4.9		
CameL (Zhou et al., 2023a)	145 (70.7)	204 (99.5)	NA	6 (2.9)	55.1	27.1	11	0.55 (0.44–0.69)	0.55 (0.42–0.71)
	101 (48.8)	199 (96.1)	NA	3 (1.4)	32.9	19.8	6.5		
KEYNOTE-021 (Awad et al., 2021)	23 (39)	55 (93)	10 (17)	1 (2)	58	34.5	24.5	0.54 (0.35–0.83)	0.71 (0.45–1.12)
	19 (31)	58 (94)	10 (16)	2 (3)	33	21.1	9.9		
RATIONALE 304 (Lu et al., 2021; Ma et al., 2024)	NA	NA	NA	NA	57.4	21.4	9.8	0.61 (0.46–0.82)	0.67 (0.48–0.95)
	NA	NA	NA	NA	36.9	20.1	7.6		
ORIENT-11 (Zhang et al., 2022; Yang et al., 2020)	164 (61.7)	265 (99.6)	16 (6)	6 (2.3)	51.9	24.2	8.9	0.482 (0.362–0.643)	0.65 (0.50–0.85)
	77 (58.8)	131 (100)	11 (8.4)	9 (6.9)	29.8	16.8	5.0		
KEYNOTE-189 (Garassino et al., 2023)	212 (52.3)	377 (93.1)	111 (27.4)	8 (2)	48.3	22	9.0	0.50 (0.42–0.60)	0.60 (0.50–0.72)
	85 (42.1)	183 (90.6)	20 (9.9)	4 (1)	19.9	10.6	4.9			
ORIENT-12 (Zhou et al., 2021)	155 (86.6)	179 (100)	26 (14.5)	8 (4.5)	44.7	NA	5.5	0.536 (0.422–0.681)	0.567 (0.353–0.909)
	148 (83.1)	178 (100)	29 (16.3)	12 (6.7)	35.4	NA	4.9		
RATIONALE 307 (Wang et al., 2021; Wang et al., 2024)	103 (85.8)	119 (99.2)	NA	1 (0.8)	73	26.1	7.7	0.45 (0.33–0.62)	0.53 (0.34–0.84)
	94 (80.3)	117 (100)	NA	3 (2.5)	50	19.4	5.5		
CameL-Sq (Ren et al., 2022; Zhou et al., 2024b)	142 (73.4)	193 (100)	23 (12)	6 (3.1)	64.8	27.4	8.5	0.37 (0.29–0.47)	0.56 (0.45–0.72)
	141 (71.9)	195 (99.5)	8 (4)	3 (1.5)	36.7	15.5	4.9		
KEYNOTE-407 (Novello et al., 2023)	159 (57.2)	266 (95.7)	58 (20.9)	12 (4.3)	62.2	17.2	8.0	0.62 (0.52–0.74)	0.71 (0.59–0.85)
	156 (55.7)	252 (90)	21 (7.5)	5 (1.8)	38.8	11.6	5.1		
CheckMate 227 Part 2 (Borghaei et al., 2023)	168 (45)	318 (85)	73 (20)	6 (2)	52	18.3	8.4	0.62 (0.52–0.73)	0.79 (0.66–0.93)
	131 (35)	291 (78)	31 (8)	0 (0)	30	14.7	5.5		
CHOICE-01 (Wang et al., 2023; Zhong et al., 2024b)	NA	NA	NA	NA	65.7	23.8	8.4	0.49 (0.39–0.61)	0.69 (0.57–0.93)
	NA	NA	NA	NA	46.2	17	5.6		
EMPOWER-Lung 3 Part 2 (Makharadze et al., 2023)	94 (30.1)	276 (88.5)	13 (4.2)	4 (1.3)	43.6	21.1	8.2	0.55 (0.44–0.68)	0.65 (0.51–0.82)
	28 (18.3)	131 (85.6)	2 (1.3)	1 (0.7)	22.1	12.9	5.5		
AK105-302 (Zhong et al., 2024a)	114 (66)	171 (99)	4 (2)	9 (5)	71	NA	7.6	0.43 (0.33–0.56)	0.55 (0.40–0.75)
	118 (67)	175 (100)	3 (2)	9 (5)	44	20.2	4.2		
ASTRUM-004 (Zhou et al., 2024a)	126 (35.2)	258 (72.1)	37 (10.3)	5 (1.4)	60.1	22.7	8.3	0.55 (0.42–0.73)	0.73 (0.58–0.93)
	58 (32.4)	114 (63.7)	9 (5)	2 (2.1)	40.2	18.2	5.7		
ASTRUM-002 (Wang et al., 2026)	142 (66)	212 (99)	33 (15)	5 (2)	52.8	NA	11	0.55 (0.43–0.69)	NA
	119 (57)	209 (99)	15 (7)	7 (3)	27.6	NA	5.6		
DOMAJOR (Laktionov et al., 2025)	NA	NA	NA	NA	50.3	NA	7.7	0.65 (0.49–0.85)	0.51 (0.35–0.73)
	NA	NA	NA	NA	27.5	14.6	5.5		

**Notes.**

TRAE treatment-related adverse effect ORR objective response rate HR hazard ratio CI confidence interval PFS progression-free survival OS overall survival

### PFS

Regarding PD-L1 inhibitors, both monotherapy (HR =0.74; 95% CI [0.66–0.83]) and combined treatment with chemotherapy (HR =0.64; 95% CI [0.58–0.72]) were associated with better PFS than chemotherapy alone (Fig. S1). Meanwhile, no difference in PFS was observed between PD-1 inhibitor monotherapy and chemotherapy alone (HR =0.77; 95% CI [0.52–1.15]). However, combination treatment was associated with longer PFS than chemotherapy alone (HR =0.53; 95% CI [0.50–0.58]; Fig. S2).

**Figure 4:**
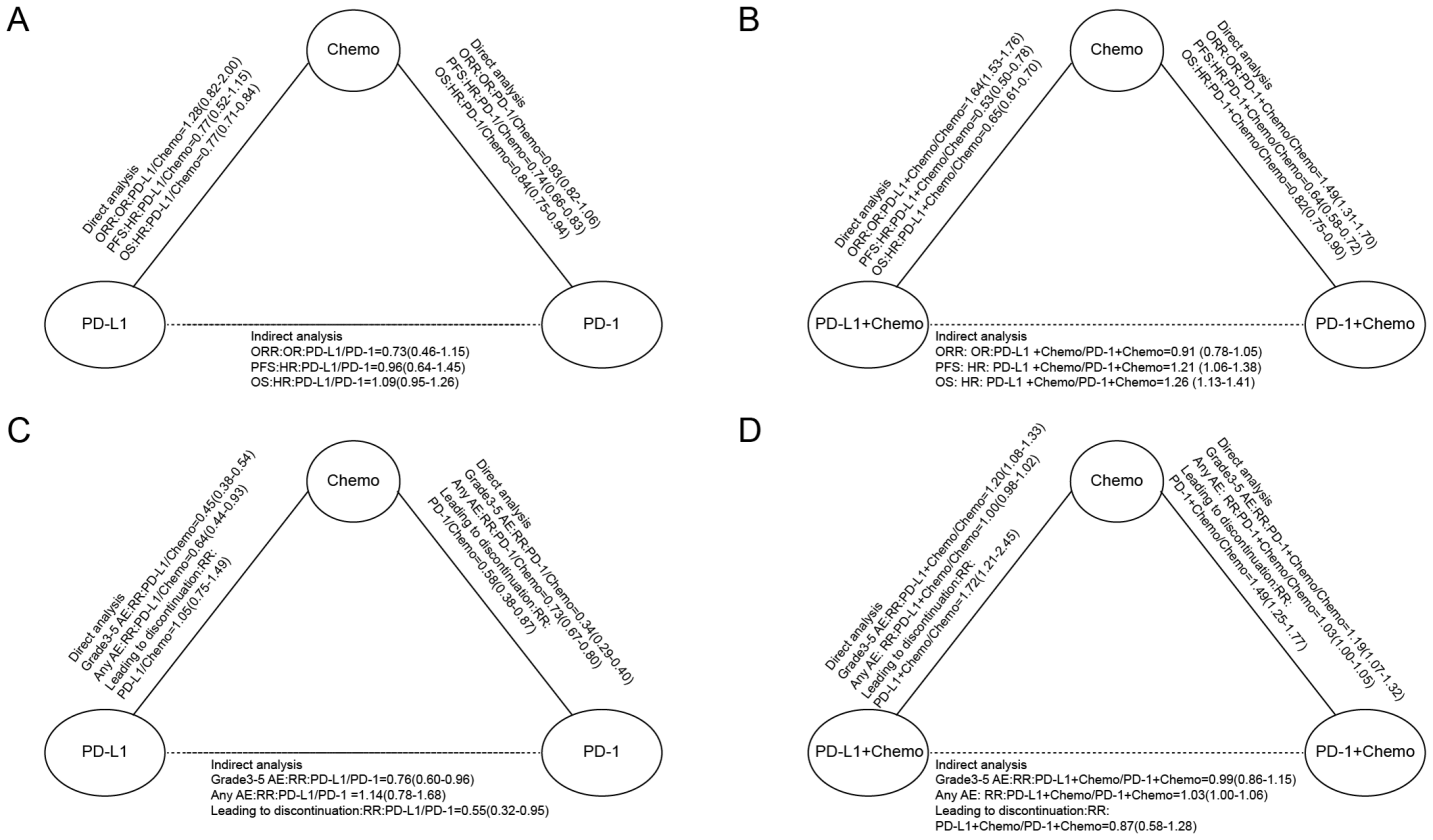
Indirect analysis comparing between PD-L1 inhibitor and PD-1 inhibitor. Indirect analysis comparing PFS, OS, ORR, the rates of grade 3–5 AEs, any-grade AEs, and AEs leading to treatment discontinuation between PD-L1 inhibitor and PD-1 inhibitor.

In indirect analysis, no difference in PFS was observed between PD-L1 inhibitor monotherapy and PD-1 inhibitor monotherapy (HR =0.96; 95% CI [0.64–1.45]; Fig. 4A). However, in the ICI–chemotherapy analysis, PD-L1 inhibitors were associated with worse PFS than PD-1 inhibitors (HR =1.21; 95% CI [1.06–1.38]; Fig. 4B).

### OS

Regarding PD-L1 inhibitors, both monotherapy (HR =0.84; 95% CI [0.75–0.94]) and combination treatment with chemotherapy (HR =0.82; 95% CI [0.75–0.90]) were associated with better OS than chemotherapy alone (Fig. S3). Similarly, PD-1 inhibitor monotherapy (HR =0.76; 95% CI [0.61–0.95]) and combination treatment with chemotherapy (HR =0.65; 95% CI [0.61–0.71]) produced better OS than chemotherapy alone (Fig. S4).

In indirect analysis, no significant difference in OS was observed between PD-L1 inhibitor monotherapy and PD-1 inhibitor monotherapy (HR =1.09; 95% CI [0.95–1.26]; Fig. 4A). However, in the ICI–chemotherapy combination group, PD-L1 inhibitors were associated with worse OS than PD-1 inhibitors (HR =1.26; 95% CI [1.13–1.41]; Fig. 4B).

### ORR

In the monotherapy trials, PD-L1 inhibitors (OR =0.93; 95% CI [0.82–1.06]) and PD-1 inhibitors (OR =1.28; 95% CI [0.82–2.00]) did not improve ORR *versus* chemotherapy alone. Concerning combination treatment, PD-L1 inhibitors plus chemotherapy (OR =1.49; 95% CI [1.33–1.60]) and PD-1 inhibitors plus chemotherapy (OR =1.64; 95% CI [1.53–1.76]) were associated with better ORR than chemotherapy alone (Figs. S5–S6).

Through indirect analysis, we observed no significant difference in ORR between ICIs and chemotherapy for both monotherapy (OR =0.73; 95% CI [0.46–1.15]) and combination treatment (OR =0.91; 95% CI [0.78–1.05]; Figs. 4A–4B).

### Grade 3–5 AEs

Treatment with PD-L1 inhibitors (RR =0.34; 95% CI [0.29–0.40]) or PD-1 inhibitors alone (RR =0.45; 95% CI [0.38–0.54]) was associated with fewer grade ≥3 AEs than chemotherapy. However, for combination treatment, both PD-L1 inhibitors (RR =1.19; 95% CI [1.07–1.32]) and PD-1 inhibitors (RR =1.20; 95% CI [1.08–1.33]) were associated with a higher risk of ≥grade 3 AEs than chemotherapy (Figs. S7–S8).

In indirect analysis, the incidence of grade ≥3 AEs was lower for PD-L1 inhibitor monotherapy than for PD-1 inhibitor monotherapy (RR =0.76; 95% CI [0.60–0.96]). Conversely, no difference was observed between PD-L1 inhibitors plus chemotherapy and PD-1 inhibitors plus chemotherapy (RR =0.99; 95% CI [0.86–1.15]; Figs. 4C–4D).

### Any-grade AEs

Through direct analysis, we found that treatment with PD-L1 inhibitors (RR = 0.73; 95% CI [0.67–0.80]) or PD-1 inhibitors alone (RR = 0.64; 95% CI [0.44–0.93]) was associated with fewer AEs than chemotherapy alone. Regarding ICI–chemotherapy combination treatment, no difference in the risk of AEs *versus* chemotherapy was detected for PD-L1 inhibitors (RR = 1.03; 95% CI [1.0–1.05]) or PD-1 inhibitors (RR = 1.00; 95% CI [0.99–1.02]; Figs. S9–S10).

In indirect analysis, no difference in the odds of AEs was observed between ICIs and chemotherapy for both monotherapy (RR = 1.14; 95% CI [0.78–1.68]) and combination treatment (RR = 1.03; 95% CI [1.00–1.06]; Figs. 4C–4D).

### AEs leading to treatment discontinuation

Regarding monotherapy, PD-L1 inhibitors were associated with fewer AEs leading to treatment discontinuation than chemotherapy (RR = 0.58; 95% CI [0.38–0.87]), whereas no difference was observed between PD-1 inhibitor monotherapy and chemotherapy (RR = 1.05; 95% CI [0.75–1.49]). Concerning combination treatment, both PD-L1 inhibitors plus chemotherapy (RR = 1.49; 95% CI [1.25–1.77]) and PD-1 inhibitors plus chemotherapy (RR = 1.72; 95% CI [1.21–2.25]) were linked to more AEs requiring treatment discontinuation than chemotherapy alone (Figs. S11–S12).

Through indirect analysis, we found that PD-L1 inhibitor monotherapy was associated with fewer AEs requiring treatment discontinuation than PD-1 inhibitor monotherapy (RR = 0.55; 95% CI [0.32–0.95]), whereas no difference in the rate of AEs leading to treatment discontinuation was observed between PD-L1 inhibitors plus chemotherapy and PD-1 inhibitors plus chemotherapy (RR = 0.87; 95% CI [0.58–1.28]; Figs. 4C–4D).

## Discussion

Immunotherapy has become an important treatment for cancer in recent years. PD-L1 and PD-1 inhibitors are the first-line treatments for advanced NSCLC lacking driver mutations. Although both PD-L1 and PD-1 inhibitors are effective, systematic reviews have reported differences in their clinical performance, and increasing numbers of studies on ICIs are being reported. Therefore, we conducted a meta-analysis to analyze the differences in efficacy and toxicity between PD-L1 and PD-1 inhibitors in the treatment of advanced NSCLC.

In 2021, a review explored the efficacy and safety of PD-1 and PD-L1 inhibitors in NSCLC. However, only 13 trials were included, including the IMpower150 study, which used bevacizumab, thereby increasing the heterogeneity of the pooled data (Brito, Camandaroba & De Lima, 2021). Our study included 28 trials, and no studies of bevacizumab were included.

Indirect analysis illustrated that PD-1 inhibitors plus chemotherapy were associated with better PFS and OS than PD-L1 inhibitors plus chemotherapy. The results were consistent with some previous meta-analyses in NSCLC (Brito, Camandaroba & De Lima, 2021; You et al., 2018; Tartarone et al., 2019). Another systematic review compared the difference in survival between PD-L1 and PD-1 inhibitors in 11,379 patients with different cancer types. The study also demonstrated that PD-1 inhibitors plus chemotherapy were associated with better PFS (HR = 0.73; 95% CI [0.56–0.96]) and OS (HR = 0.75; 95% CI [0.65–0.86]) than PD-L1 inhibitors plus chemotherapy. This finding might be related to the synergistic effects of chemotherapy and immunosuppressants. The cytotoxicity of chemotherapy leads to tumor antigen exposure and increased cross-presentation of dendritic cell antigens, thereby increasing effector T cell counts, inhibiting regulatory T cells, and enhancing immunity (Medler, Cotechini & Coussens, 2015; Galluzzi et al., 2015; Roselli et al., 2013; Bracci et al., 2014). In addition, PD-1 inhibitors bind to both PD-L1 and PD-L2, whereas PD-L1 inhibitors only bind to PD-L1, which is an important factor in inhibiting T cell activity (Taube et al., 2014; Callahan & Wolchok, 2013; Keir et al., 2008). The inhibition of both PD-L2 and PD-L1 can lead to synergistic anti-tumor effects (Brown et al., 2003). However, regarding small cell lung cancer (SCLC), no difference in PFS (HR = 1.10; 95% CI [0.88–1.37]) or OS (HR = 0.99; 95% CI [0.77–1.23]) was recorded between PD-1 inhibitors plus chemotherapy and PD-L1 inhibitors plus chemotherapy (Yu et al., 2022). A potential reason might be the different immune microenvironments of SCLC and NSCLC; for example, PD-L1 expression is usually low or deficient in SCLC (Iams, Porter & Horn, 2020). We found no differences between the two groups regarding the rates of any-grade AEs, AEs leading to treatment interruption, and grade ≥3 AEs. One possible reason is that the AEs of chemotherapy combined with immunosuppressants are mainly dominated by those caused by chemotherapy.

Concerning monotherapy, we found no differences in PFS, OS, and ORR between PD-L1 inhibitors and PD-1 inhibitors. AEs leading to treatment discontinuation and grade ≥3 AEs were more frequent for PD-1 inhibitors than PD-L1 inhibitors. This might be attributable to the fact that PD-L1 is the main driver of T cell inhibition, and most cancers exhibit higher PD-L1 expression than PD-L2 expression (Takamochi et al., 2022; Inoue et al., 2016). In addition, studies of immunotherapy *versus* chemotherapy all enrolled PD-L1-positive populations. These findings could explain why PD-1 inhibitors did not prove more effective than PD-L1 inhibitors despite acting on both PD-L1 and PD-L2. However, because PD-1 inhibitors target both PD-L1 and PD-L2, the incidence of AEs leading to treatment discontinuation and and grade ≥3 AEs was higher for PD-1 inhibitors than for PD-L1 inhibitors.

Our study had multiple limitations. First, the numbers of RCTs focused on PD-L1 and PD-1 were different. This is related to the low number of PD-L1 drugs used to treat NSCLC. Second, we did not conduct subgroup analysis based on the PD-L1 expression status, pathological type, or other variables. PD-L1 levels and pathological types can affect the efficacy of immunotherapy, but some studies did not report subgroup data based on these factors. Third, several included studies used different treatment strategies and inconsistent durations of follow-up, but all RCTs were confirmed to have a low risk of bias after the quality assessment. Therefore, the pooled results of the meta-analysis should be reliable. Finally, our findings were obtained from indirect comparisons rather than head-to-head prospective comparisons. Unfortunately, such prospective studies have not been conducted, which makes this indirect comparison critical to current clinical needs. Our findings provide evidence for the use of ICIs in NSCLC.

## Conclusions

The current meta-analysis, which included 28 high-quality trials involving 14,758 patients, demonstrated that PD-1 inhibitors plus chemotherapy may provided superior efficacy compared with PD-L1 inhibitors plus chemotherapy in the first-line treatment of advanced NSCLC, whereas their AEs were similar. Regarding monotherapy, only the rate of AEs leading to treatment discontinuation and grade ≥3 AEs differed between PD-1 inhibitors and PD-L1 inhibitors.

##  Supplemental Information

10.7717/peerj.21402/supp-1 Supplemental Information 1 Data and statistical steps for indirect comparison

10.7717/peerj.21402/supp-2 Supplemental Information 2 PRISMA checklist.

10.7717/peerj.21402/supp-3 Supplemental Information 3 Audience of the study.

10.7717/peerj.21402/supp-4 Supplemental Information 4 Original data included in this investigation. Detailed primary data collected from 28 clinical research studies.

10.7717/peerj.21402/supp-5 Supplemental Information 5 Direct comparison of PD-1 or PD-L1 with chemotherapy and direct comparison of PD-1 or PD-L1 combined chemotherapy with chemotherapy Twelve charts detail the differences in efficacy and safety between PD-1 or PD-L1 and chemotherapy, and compare the differences in efficacy and safety between PD-1 or PD-L1 combined chemotherapy and chemotherapy

## List of Abbreviations

AEs: adverse effects
CI: confidence interval
HR: hazard ratio
OR: odds ratio
RR: risk ratio
ICIs: immune checkpoint inhibitors
NSCLC: non-small cell lung cancer
OS: overall survival
ORR: objective response rate
ORRs: overall response rates
PFS: progression-free survival
SCLC: small cell lung cancer
RCTs: randomized controlled trials

## Additional Information and Declarations

### Data Availability

The following information was supplied regarding data availability: This is a Systematic review/Meta-analysis.
