# Supplementary material for: Efficacy and safety of Programmed Death Ligand 1 inhibitors versus Programmed Death 1 inhibitors in the first-line treatment of advanced non-small cell lung cancer: a meta-analysis of randomized controlled trials
Source: PeerJ. 2026 Jul 7;14:e21402. doi: 10.7717/peerj.21402 (PMC13353234; doi:10.7717/peerj.21402)
Supplement: Supplemental Information 3 [file peerj-14-21402-s003.doc]

The audience in our study is the first-line treatment of patients with advanced, treatment-naïve non-small cell lung cancer
