# Supplementary material for: Efficacy and safety of Programmed Death Ligand 1 inhibitors versus Programmed Death 1 inhibitors in the first-line treatment of advanced non-small cell lung cancer: a meta-analysis of randomized controlled trials
Source: PeerJ. 2026 Jul 7;14:e21402. doi: 10.7717/peerj.21402 (PMC13353234; doi:10.7717/peerj.21402)
Supplement: Supplemental Information 5 — Twelve charts detail the differences in efficacy and safety between PD-1 or PD-L1 and chemotherapy, and compare the differences in efficacy and safety between PD-1 or PD-L1 combined chemotherapy and chemotherapy [file peerj-14-21402-s005.docx]

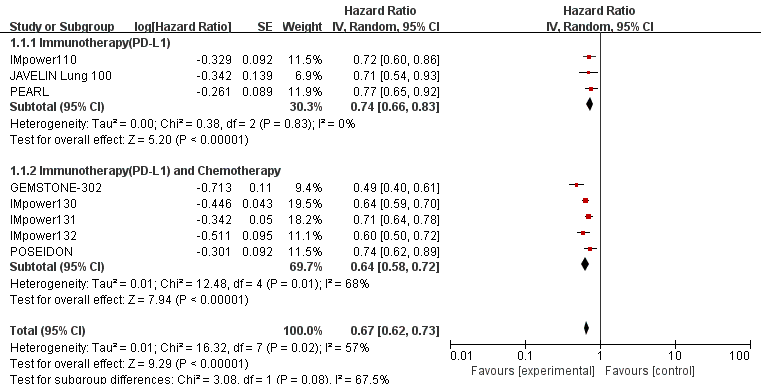


**Figure S1.** Forest plot comparing progression-free survival between PD-L1 inhibitor monotherapy and PD-L1 inhibitors plus chemotherapy. CI, confidence interval. Note: Jassem J et al. (2021), Reck M et al. (2024), Lu S et al. (2025), Zhou C et al. (2023), Zhou C et al. (2024), West H et al. (2019), Jotte R et al. (2020), Nishio M et al. (2021), Johnson ML et al. (2023).


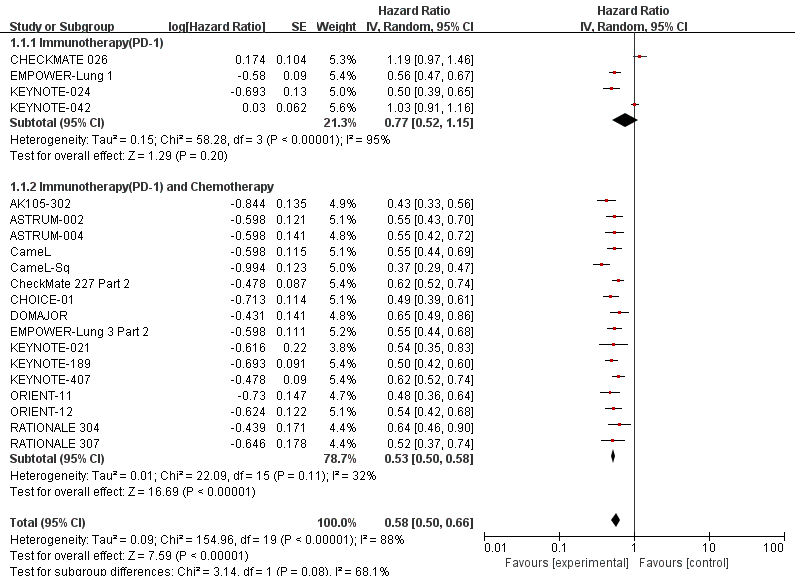


**Figure S2.** Forest plot comparing progression-free survival between PD-1 inhibitor monotherapy and PD-1 inhibitors plus chemotherapy. CI, confidence interval. Note: Carbone DP et al. (2017), Özgüroğlu M et al. (2023), Kilickap S et al. (2024), Reck M et al. (2021), de Castro G et al. (2023), De Castro G et al. (2021), Zhong H et al. (2024), Wang L et al. (2025), Zhou C et al. (2024), Zhou C et al. (2023), Ren S et al. (2022), Zhou C et al. (2024), Borghaei H et al. (2023), Wang Z et al. (2023), Zhong J et al. (2024), Laktionov K et al. (2025), Makharadze T et al. (2023), Awad MM et al. (2021), Garassino MC et al. (2023), Novello S et al. (2023), Zhang L et al. (2022), Zhou C et al. (2021), Lu S et al. (2021), Ma Z et al. (2024), Wang J et al. (2021), Wang Z et al. (2024).


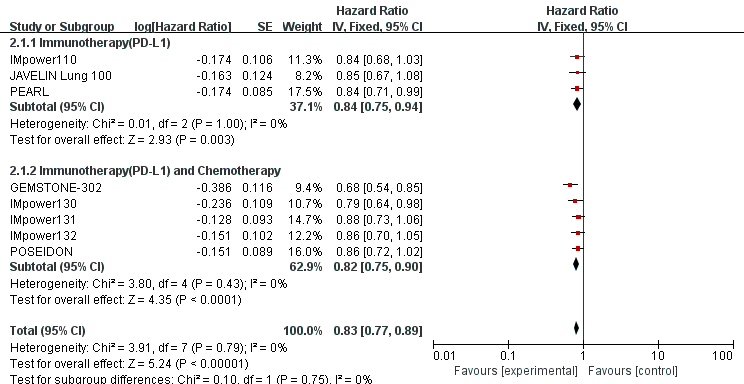


**Figure S3.** Forest plot comparing overall survival between PD-L1 inhibitor monotherapy and PD-L1 inhibitors plus chemotherapy. CI, confidence interval. Note: Jassem J et al. (2021), Reck M et al. (2024), Lu S et al. (2025), Zhou C et al. (2023), Zhou C et al. (2024), West H et al. (2019), Jotte R et al. (2020), Nishio M et al. (2021), Johnson ML et al. (2023).


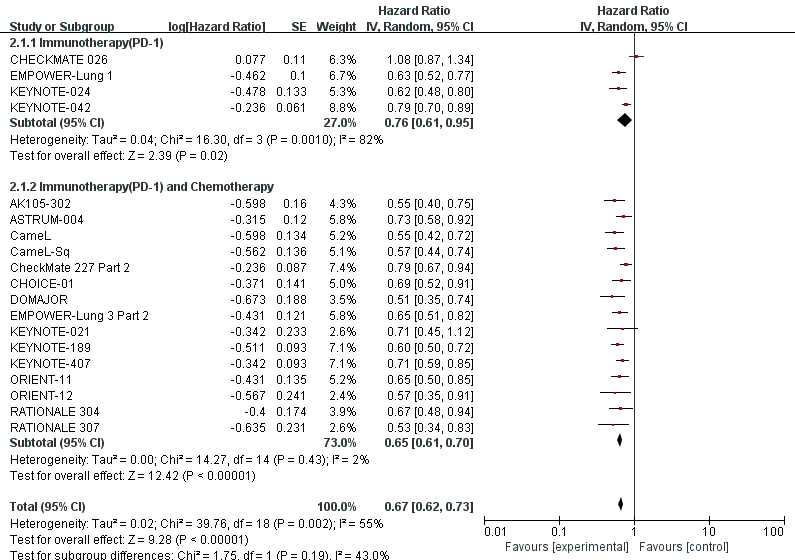


**Figure S4.** Forest plot comparing overall survival between PD-1 inhibitor monotherapy and PD-1 inhibitors plus chemotherapy. CI, confidence interval. Note: Carbone DP et al. (2017), Özgüroğlu M et al. (2023), Kilickap S et al. (2024), Reck M et al. (2021), de Castro G et al. (2023), De Castro G et al. (2021), Zhong H et al. (2024), Wang L et al. (2025), Zhou C et al. (2024), Zhou C et al. (2023), Ren S et al. (2022), Zhou C et al. (2024), Borghaei H et al. (2023), Wang Z et al. (2023), Zhong J et al. (2024), Laktionov K et al. (2025), Makharadze T et al. (2023), Awad MM et al. (2021), Garassino MC et al. (2023), Novello S et al. (2023), Zhang L et al. (2022), Zhou C et al. (2021), Lu S et al. (2021), Ma Z et al. (2024), Wang J et al. (2021), Wang Z et al. (2024).


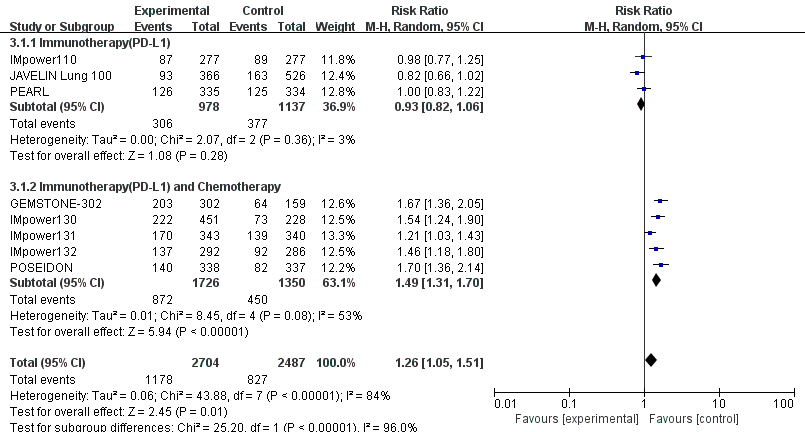


**Figure S5.** Forest plot comparing the objective response rate between PD-L1 inhibitor monotherapy and PD-L1 inhibitors plus chemotherapy. CI, confidence interval. Note: Jassem J et al. (2021), Reck M et al. (2024), Lu S et al. (2025), Zhou C et al. (2023), Zhou C et al. (2024), West H et al. (2019), Jotte R et al. (2020), Nishio M et al. (2021), Johnson ML et al. (2023).


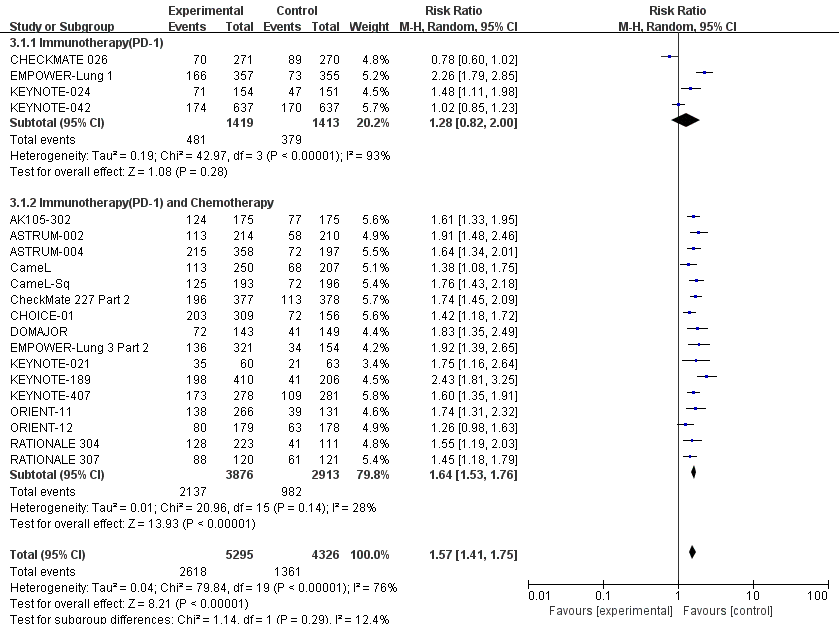


**Figure S6.** Forest plot comparing the objective response rate between PD-1 inhibitor monotherapy and PD-1 inhibitors plus chemotherapy. CI, confidence interval. Note: Carbone DP et al. (2017), Özgüroğlu M et al. (2023), Kilickap S et al. (2024), Reck M et al. (2021), de Castro G et al. (2023), De Castro G et al. (2021), Zhong H et al. (2024), Wang L et al. (2025), Zhou C et al. (2024), Zhou C et al. (2023), Ren S et al. (2022), Zhou C et al. (2024), Borghaei H et al. (2023), Wang Z et al. (2023), Zhong J et al. (2024), Laktionov K et al. (2025), Makharadze T et al. (2023), Awad MM et al. (2021), Garassino MC et al. (2023), Novello S et al. (2023), Zhang L et al. (2022), Zhou C et al. (2021), Lu S et al. (2021), Ma Z et al. (2024), Wang J et al. (2021), Wang Z et al. (2024).


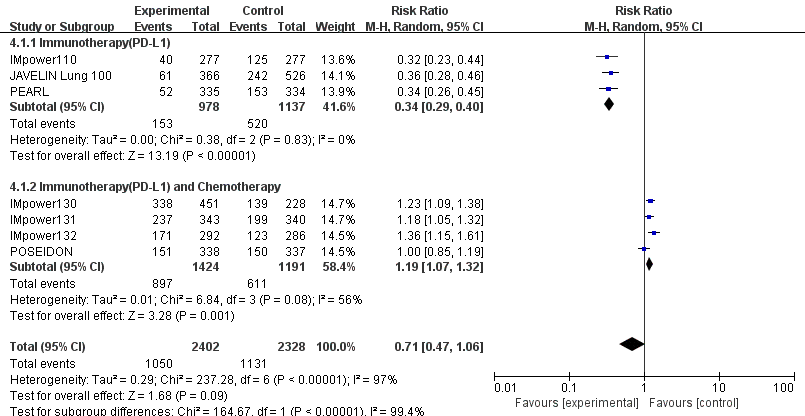


**Figure S7** Forest plot of comparing the rates of grade 3–5 adverse effects between PD-L1 inhibitor monotherapy and PD-L1 inhibitors plus chemotherapy. CI, confidence interval. Note: Jassem J et al. (2021), Reck M et al. (2024), Lu S et al. (2025), West H et al. (2019), Jotte R et al. (2020), Nishio M et al. (2021), Johnson ML et al. (2023).


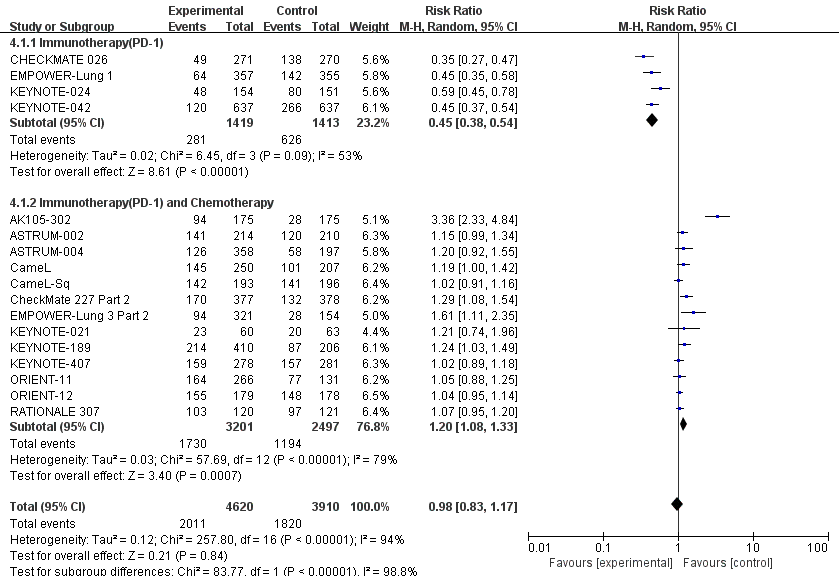


**Figure S8.** Forest plot comparing the rates of grade 3–5 adverse effects between PD-1 inhibitor monotherapy and PD-1 inhibitors plus chemotherapy. CI, confidence interval. Note: Carbone DP et al. (2017), Özgüroğlu M et al. (2023), Kilickap S et al. (2024), Reck M et al. (2021), de Castro G et al. (2023), De Castro G et al. (2021), Zhong H et al. (2024), Wang L et al. (2025), Zhou C et al. (2024), Zhou C et al. (2023), Ren S et al. (2022), Zhou C et al. (2024), Borghaei H et al. (2023), Makharadze T et al. (2023), Awad MM et al. (2021), Garassino MC et al. (2023), Novello S et al. (2023), Zhang L et al. (2022), Zhou C et al. (2021), Wang J et al. (2021), Wang Z et al. (2024).


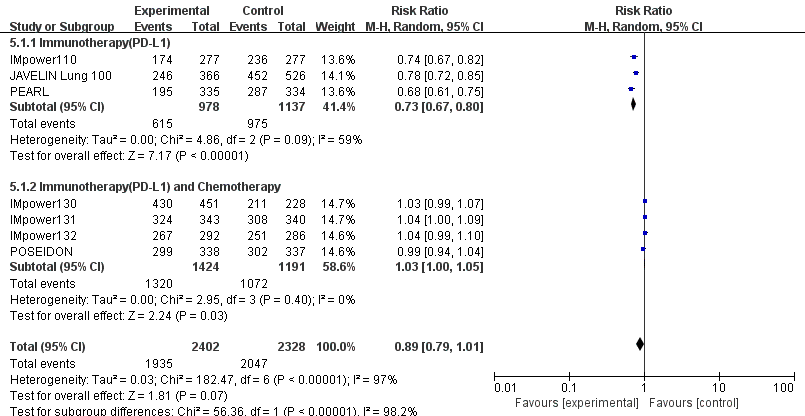


**Figure S9.** Forest plot comparing the rates of any-grade adverse effects between PD-L1 inhibitor monotherapy and PD-L1 inhibitors plus chemotherapy. CI, confidence interval. Note: Jassem J et al. (2021), Reck M et al. (2024), Lu S et al. (2025), West H et al. (2019), Jotte R et al. (2020), Nishio M et al. (2021), Johnson ML et al. (2023)


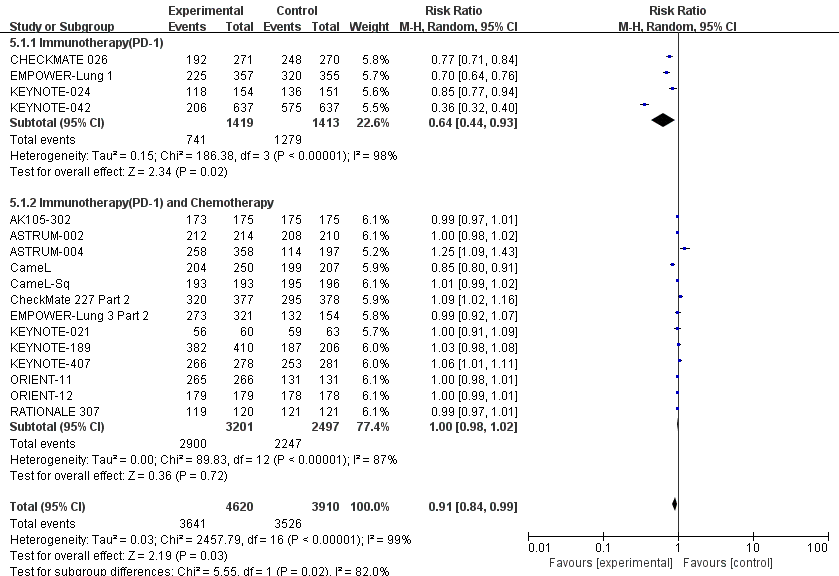


**Figure S10.** Forest plot comparing the rates of any-grade adverse effects between PD-1 inhibitor monotherapy and PD-1 inhibitors plus chemotherapy. CI, confidence interval. Note: Carbone DP et al. (2017), Özgüroğlu M et al. (2023), Kilickap S et al. (2024), Reck M et al. (2021), de Castro G et al. (2023), De Castro G et al. (2021), Zhong H et al. (2024), Wang L et al. (2025), Zhou C et al. (2024), Zhou C et al. (2023), Ren S et al. (2022), Zhou C et al. (2024), Borghaei H et al. (2023), Makharadze T et al. (2023), Awad MM et al. (2021), Garassino MC et al. (2023), Novello S et al. (2023), Zhang L et al. (2022), Zhou C et al. (2021), Wang J et al. (2021), Wang Z et al. (2024).


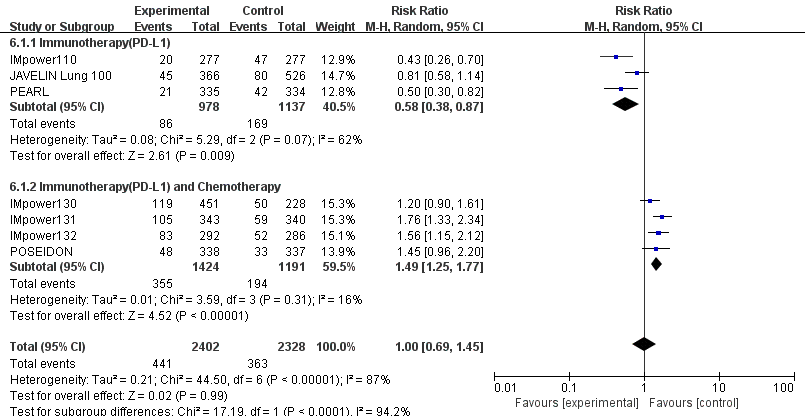


**Figure S11.** Forest plot comparing the rates of adverse effects leading to treatment discontinuation between PD-L1 inhibitor monotherapy and PD-L1 inhibitors plus chemotherapy. CI, confidence interval. Note: Jassem J et al. (2021), Reck M et al. (2024), Lu S et al. (2025), West H et al. (2019), Jotte R et al. (2020), Nishio M et al. (2021), Johnson ML et al. (2023).


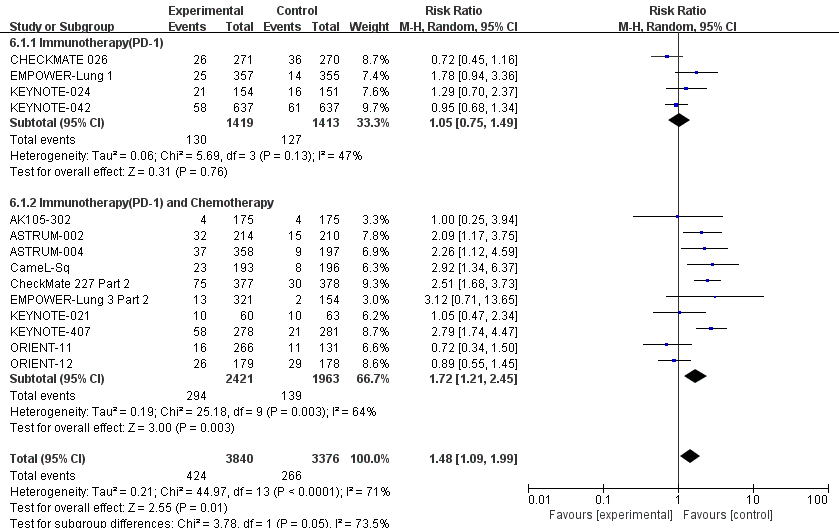


**Figure S12.** Forest plot comparing the rates of adverse effects leading to treatment discontinuation between PD-1 inhibitor monotherapy and PD-1 inhibitors plus chemotherapy. CI, confidence interval. Note: Carbone DP et al. (2017), Özgüroğlu M et al. (2023), Kilickap S et al. (2024), Reck M et al. (2021), de Castro G et al. (2023), De Castro G et al. (2021), Zhong H et al. (2024), Wang L et al. (2025), Zhou C et al. (2024), Ren S et al. (2022), Zhou C et al. (2024), Borghaei H et al. (2023), Makharadze T et al. (2023), Awad MM et al. (2021), Novello S et al. (2023), Zhang L et al. (2022), Zhou C et al. (2021).
